# Supplementary material for: Green gentrification in European and North American cities
Source: Nat Commun. 2022 Jul 2;13:3816. doi: 10.1038/s41467-022-31572-1 (PMC9250502; doi:10.1038/s41467-022-31572-1)
Supplement: Supplementary file 1 — Supplementary Information [file 41467_2022_31572_MOESM1_ESM.pdf]

SUPPLEMENTARY INFORMATION

Green gentrification in European and North American Cities

Supplementary Note

A brief literature review of existing ways that gentrification is conceptualized and operationalized:

It is important to briefly ground this study in the existing definition(s) and manifestations of gentrification and its commonly identified drivers. Since the term gentrification debuted in academic and social debates over half a century ago with Ruth Glass' classic study of London, there has been extensive deliberation around how, exactly, it should be defined and measured. Major debates around defining the term have centered on the importance of production-side versus consumption-side explanations of gentrification, the characteristics and preferences of the middle class and if/how displacement relates to gentrification, among others (Smith and Williams 1986; Slater 2011).

Some of these debates have petered out. For example, there is general agreement that structural forces (production) as advocated by Smith (1979) and cultural dynamics (consumption) as proposed by Ley (1981) should be considered as interdependent in gentrification processes (Hamnett 1991; Clark, 2005; Lees et al. 2008). Other debates, such as if and how gentrification displaces or replaces original residents (Marcuse 1985; Newman and Wyly 2006; Easton et al. 2020; Elliot-Cooper, Hubbard, and Lees, 2020) or the planetary or global dimensions of gentrification (Janoschka et al. 2014; Lees et al. 2015), remain ongoing. Debates aside, many scholars concur with the core processes described by Ruth Glass in coining the term gentrification in late 1950s London: the social, economic, and physical transformation of de- or under-valued working class districts into middle and upper class enclaves, replacing the original occupants (Glass 1964).

Most of these definitional discussions are related to how to conceptualize and prioritize the drivers of gentrification, and how the concept, more broadly speaking, might be operationalized in terms of measurement. In economics, gentrification is often studied by examining the location – and change of location or location choice – of higher income residents (Brueckner and Rosenthal, 2009; Kahn and Walsh, 2004). In planning and geography, general quantitative approaches to understanding gentrification employ a set of socioeconomic and real estate indicators – including income, ethnicity, race, education, occupational status, age, tenure status and housing/rental prices (Hammel and Wyly 1996; Glick 2008) – at the census tract or neighborhood level. An increase through time in median income, formal education, white residents, housing values, rental prices and capital investment and decreases in poor and less formally educated residents are some indicators that are broadly correlated to processes of gentrification (Atkinson 2000; Freeman and Braconi 2004).

While there is an emerging effort to develop innovative and higher resolution data reflective of gentrification trends using, for example, google street view (Hwang and Sampson 2014), these approaches are not a panacea for empirical studies of gentrification as they do not deviate much from the prior quantitative trends based on public socioeconomic data (Zook et al. 2019). In all, the variables used in quantitative studies have been fairly stable since the 1990s. Variation is generally based on available data. Thus, we employed the widest set of these stable variables for which data could be attained across most of our study cities.

Supplementary Methods

In preparing the data for analysis, we used a number of data sources to acquire demographic data (see Supplemementary Table 1) and real estate data. For each city included in the analysis, we sought data at the finest geographic scale available. The majority of data came from national censuses, and thus the unit of analysis for demographic data corresponded to the census tract, area, or other similar geographic unit for which data in the corresponding country is released. For each country, we searched for available demographic data at the level of these small geographic units that corresponded to constructs commonly used to measure gentrification (i.e., level of education, income or other measure of socioeconomic status, race/ethnicity or migration status, special variables relative to the local context, and where available data on cost of housing). See Supplementary Table 2 for a list of variables acquired in each country.

In addition to variables used to measure gentrification, we collected basic population data at the same geographic scale which were included in the analyses and which was used to calculate the relative measure of each variable (see below). The data sources used for the project; variables, geographic units and timeframes used; and the processing of demographic data is described below.

Supplementary Table 1. Data sources consulted, organized by country

**Cities (Country)**	**Demographic Data Source**	**Unit of Analysis**
Lyon and Nantes (France)	INSEE (1999; 2006-2013)	IRIS
Atlanta, Austin, Boston, Cleveland, Denver, Detroit, Louisville, Milwaukee, Philadelphia, Portland, San Francisco, Seattle,	US Census (1990-2010), American Community Survey (2006-2010 and 2012-2016), Data standardized to 2010 census tracts purchased from GeoLytics (Neighborhood Change database)	Census Tract

Washington DC (US)		
Calgary, Montreal and Vancouver (Canada)	Canadian Census (1991, 1996, 2001, 2006, 2011, 2016)	Enumeration areas (1991, 1996); Dissemination areas (post 1996)
Palermo, and Milan (Italy)	Italian Census from ISTAT (1991, 2001, 2011)	Sezione di censimento (1991, 2001), Aree di censimento (2011)
Barcelona (Spain)	For age, profession, education, vulnerability: Instituto Nacional de Estadística (1991, 2001, 2011) and register of inhabitants (1996 or 1997, 2006, 2016)<br><br>For socioeconomic data and rent value data: Barcelona Town Council Statistics office (1991, 1992, 1996, 2000, 2001, 2006, 2007, 2008, 2009, 2010, 2011, 2012, 2013, 2014, 2015, 2016)	Census tract, Small Research Area or Neighborhood depending on year and variable
Sheffield, and Bristol (UK)	UK Census (1991, 2001, 2011)	Enumeration Districts (1991), Output Area (2001, 2011)
Edinburgh (UK)	UK Census (1991, 2001, 2011)	Output Areas
Amsterdam (The Netherlands)	Municipal Research, Information and Statistics Office (1997-2017), Dutch national Statistics Netherlands (1997-2007, 2001, 2006, 2010),	Wijk
Copenhagen (Denmark)	Danish Statistical Office (yearly from 1990 to 2016)	Roder
Stuttgart (Germany)	For age, vulnerability, housing ownership: Department for Statistics – City of Stuttgart (1990 or 1991, 2000, 2015)	Stadtteil (Sub-District)
Vienna (Austria)	For age, education, profession, socioeconomic status, and vulnerability: City of Vienna Austrian Public Data	Zählgebiet (Sub-Districts)

	Portal (1991, 2001, 2014 or 2015 or 2016)	
Dublin (Ireland)	Ireland Census (1996, 2002, 2006, 2011, 2016)	Electoral Divisions (called SAPS in 1996)
Valencia (Spain)	For age, profession, education, vulnerability: Instituto Nacional de Estadística censuses (1991, 2001, 2011) and register of inhabitants (1996, 2006, 2016).<br><br>For rent value data: Statistics office at the Valencia Town Council. Statistics Yearbooks (1993, 1996, 2001, 2002, 2006, 2011, 2012, 2013, 2014, 2015, 2016)	Census tract or neighborhood depending on year and variable

Variables

As the type of data collected by national censuses and other data consulted vary by local context, we revised available variables in the case of each city and balanced the availability of data with the two constructs we sought to measure and the local context so that the data sets for each city/country could be as comparable as possible. We sought to find variables representing the following gentrification constructs for each city which are constructs traditionally used or referred to in the gentrification literature: 1) social vulnerability: population with at least a university education, population with low income (relative to the city-wide median income) or vulnerable racial or immigration origin, population with high income (considered to be higher than 125% above the city-wide median income), adult population working in professional class jobs, race or ethnicity; and 2) housing cost: area average home sale and area average rental value. In many cases, these exact variables were not available and in these cases, we sought to find the best possible match for the constructs. See Supplementary Table 2 below for the final list of variables by city.

First, we considered "social vulnerability" as one of the constructs needed to measure gentrification. We included it because measuring change in the proportion of socially vulnerable population over time in a given neighborhood or small area may indicate whether socially vulnerable populations may be experiencing expulsion from the area. We counted as aspects of social vulnerability either household or individual socioeconomic indicators (where individuals with lower levels of education, lower incomes, or lower social classes were considered to be more socially vulnerable than others), measures of race/ethnicity (where populations of minority races or ethnicities may be considered more socially vulnerable than the dominate race or ethnicity), and measures of immigration or migration which may be used in lieu of race/ethnicity in cities in which race/ethnicity is not measured in the data or where recent immigration or place of birth is more important for determining level of social vulnerability than race or ethnicity. To identify which variables to use to measure this construct, we consulted existing academic literature focusing on each local context and local grey literature reporting on social or health inequality to get a sense of the dimensions by

which social vulnerability had been reported. We then selected the best variables available at the appropriate geographic scale to fit with the local context regarding social vulnerability.

Measures of socioeconomic social vulnerability also varied by local context. Socioeconomic status was measured using a selection of related variables such as highest level of education, household income, employment status and professional category.

As the second construct, we measured cost of housing as rapidly increasing housing prices, both rental prices and purchasing prices, are an indicator of gentrification. However, this data was often not available or was available from a different source and/or at a different geographic scale than data from the local census. In the final gentrification score calculation, only change in rental price was used as this was the most universal variable.

Supplementary Table 2. Specific and detailed variables used for each country/city when calculating the final gentrification score

Table S1 – APPROXIMATELY YEAR 2000

City	Year represented	Vulnerability	Education	Income	Real estate information	Poverty	Professionals
**Atlanta**(Georgia, US)<br>**Austin** (Texas, US)<br>**Baltimore** (Maryland, US)<br>**Boston** (Massachusetts, US)<br>**Cleveland**(Ohio, US)<br>**Denver** (Colorado, US)<br>**Detroit**(Michigan, US)<br>**Louisville**(Kentucky, US)<br>**Milwaukee**(Wisconsin, US)<br>**Philadelphia**(Pennsylvania, US)<br>**Portland**(Oregon, US)<br>**San Francisco**(California, US)<br>**Seattle**(Washington, US)<br>**Washington DC** (District of Columbia, US)	2000	**PC_NVULN00T** *"Percentage of non-Hispanic White residents (**NVULN00T/VULTOT00T**)"*<br><br>**NVULN00T:**<br>*Non-Hispanic white only*<br><br>**VULTOT00T:**<br>*Total number of people for race/ethnicity variable*<br><br>***<br>**Data source:** *US Census (1990-2010), American Community Survey (2006-2010 and 2012-2016), Data standardized to 2010 census tracts purchased from GeoLytics (Neighborhood Change database)*<br><br>***<br>**Unit of Analysis**: *Census Tract*	**PC_UNI00T** *"Percentage of residents with university degree or higher (**UNI10T/EDUTOT00T**)"*<br><br>**UNI00T:**<br>*people with university degree or higher*<br><br>**EDUTOT00T:**<br>*Total number of people for education variables*<br><br>***<br>**Data source:** *US Census (1990-2010), American Community Survey (2006-2010 and 2012-2016), Data standardized to 2010 census tracts purchased from GeoLytics (Neighborhood Change database)*<br><br>***<br>**Unit of Analysis**: *Census Tract*	**PC_HISES00T** *"Percentage of residents with high income (**HISES00T/SESTOT00T**)"*<br><br>**HISES00T:**<br>*Household income categories, adjusted for each city such that low SES = those in categories above 125% of the median for the city for the year*<br><br>**SESTOT00T**:<br>*Used aggregate of all categories rather than provided denominator due to error because of the standardization of the census tracts to 2010 areas according to suggestions from Geolytics*<br><br>***<br>**Data source:** *US Census (1990-2010), American Community Survey (2006-2010 and 2012-2016), Data standardized to 2010 census tracts purchased from*	**PC_HRENT00T** *"Percent age of households paying above median value in rent (**HRENT00T/RENT00T**)"*<br><br>***HRENT00T:***<br>*Rental households paying above the median value in rent, adjusted for each city and year to the median rent for the city for the year*<br><br>**RENT00T:**<br>*Used aggregate of all rent categories rather than provided denominator due to error because of the standardization of the census tracts to 2010 areas according to suggestions from Geolytics*<br><br>***<br>**Data source:** *US Census (1990-2010), American Community Survey (2006-2010 and 2012-2016), Data standardized to 2010 census tracts purchased from GeoLytics*	**PC_NPOV00T** *"Percentage of residents not below poverty level (**POV00T/POVTOT00T**)"*<br><br>**POV00T**:<br>*Number of people determined to be below poverty level*<br><br>**POVTOT00T**:<br>*Total number for which poverty is determined*<br><br>***<br>**Data source:** *US Census (1990-2010), American Community Survey (2006-2010 and 2012-2016), Data standardized to 2010 census tracts purchased from GeoLytics (Neighborhood Change database)*<br><br>***<br>**Unit of Analysis**: *Census Tract*	**PC_PROF00T** *"Percentage of residents with professional occupation (**PROF00T/PROFTOT00T**)"*<br><br>**PROF00T:**<br>*Aggregate of two categories: 1) Professional, scientific, and management, and administrative and waste management services: - Professional, scientific, and technical services and 2) Professional, scientific, and management, and administrative and waste management services: - Management of companies and enterprises*<br><br>**PROFTOT00T:**<br>*Total number of people asked about occupation*<br><br>***<br>**Data source:** *US Census (1990-2010), American Community Survey (2006-2010 and 2012-2016), Data standardized to 2010 census tracts purchased from*

				GeoLytics (Neighborhood Change database)<br><br>***<br>**Unit of Analysis***: Census Tract*	*(Neighborhood Change database)*<br><br>***<br>**Unit of Analysis***: Census Tract*		*GeoLytics (Neighborhood Change database)*<br><br>***<br>**Unit of Analysis***: Census Tract*
**Calgary**(Canada)<br>**Montreal**(Canada)<br>**Vancouver**(Canada)	2001	**PCNVULN01T**<br>*“Percentage of Non-vulnerable community* ***(VISMIN01T/VISMINT0T01T”*** *)*<br><br>**VISMIN01T:**<br>*Total population in private households by visible minority (Black, Arab, S. Asian and multiple visible minorities).*<br><br>**VISMINT0T01T:**<br>*Total population by visible minority*<br><br>***<br>**Data source:** *Canadian Census (1991, 1996, 2001, 2006, 2011, 2016)*<br><br>***<br>**Unit of Analysis***: Enumeration areas (1991, 1996); Dissemination areas (post 1996)*	**PCUNI01T**<br>*“Percentage of residents with university degree or higher* ***(UNI01T/EDUTOT01T)****”*<br><br>**UNI01T:**<br>*Population 20+ with university degree or higher*<br><br>**EDUTOT01T:**<br>*Total number of people for education variables per EA or DA*<br><br>***<br>**Data source:** *Canadian Census (1991, 1996, 2001, 2006, 2011, 2016)*<br><br>***<br>**Unit of Analysis***: Enumeration areas (1991, 1996); Dissemination areas (post 1996)*	**PC_HISES01T**<br>*“Percentage of residents with high income* ***(HISES01T/SESTOT01T)***”<br><br>**HISES01T:**<br>*High SES/Total for this variable (125% above city median household income)*<br><br>**SES01T:**<br>*Total SES (Household income - all private households)*<br><br>***<br>**Data source:** *Canadian Census (1991, 1996, 2001, 2006, 2011, 2016)*<br><br>***<br>**Unit of Analysis***: Enumeration areas (1991, 1996); Dissemination areas (post 1996)*	**PC_HRENT01T**<br>*“Percentage of households paying above median value in rent(****HRENT01T/RENT01T****)”*<br><br>**HRENT01T**<br>*Rental households paying above the median value in rent, adjusted for each city and year to the median rent for the city for the year*<br><br>**RENT01T:**<br>*Total number of renters*<br><br>***<br>**Data source:** *Canadian Census (1991, 1996, 2001, 2006, 2011, 2016)*<br><br>***<br>**Unit of Analysis***: Enumeration areas (1991, 1996); Dissemination areas (post 1996)*	**N/A**	**PC_PROF01T**<br>*“Percentage of residents with professional occupation* ***(PROF01T/PROFTOT01T)***<br><br>**PROF01T:**<br>*People working a professional occupation; 20+ categories*<br><br>**PROFTOT01T:**<br>*Total occupation/occupation total denominator*<br><br>***<br>**Data source:** *Canadian Census (1991, 1996, 2001, 2006, 2011, 2016)*<br><br>***<br>**Unit of Analysis***: Enumeration areas (1991, 1996); Dissemination areas (post 1996)*
**Lyon**<br>(France)<br>**Nantes**<br>(France)	1999	**PC_NVULN99**<br>*“Percentage of non-vulnerable residents (1-* ***VULN99T/VULTOT99T)****”*<br><br>**VULN99T:**<br>*Vulnerable community (specify)-smallest geography /PLACE OF BIRTH - Foreign (1999); Number of immigrants (2006-2013)*<br><br>**VULTOT99T:**<br>Total population, smallest geography<br><br>***	**PC_UNI99**<br>*“Percentage of residents with university degree or higher* ***(UNI99T/EDUTOT99T)****”*<br><br>**UNI99T:**<br>*People with a university degree or higher / Senior Degrees*<br><br>**EDUTOT99T:**<br>*Education total, smallest geography / Population 15 years and over by sex, age*	**MEDINC99T:**<br>*Median income, smallest geography*<br><br>***<br>**Data source:** *INSEE (1999; 2006-2013)*<br><br>***<br>**Unit of Analysis***: IRIS*	**N/A**	**PC_NPOV99**<br>*“Percentage of social housing primary residences* *<u>not</u>* *below poverty level (****1-HLMV99T/HHTOT99T****)”*<br><br>**HLMV99T:**<br>*Number of social housing , primary residences rented empty - an empty rented accommodation HLM*<br><br>**HHTOT99T:**<br>*Total number of primary residences (denominator)/ Number of main residences*	**PC_PROF99**<br>*“Percentage of residents with professional occupation* ***(PROF99T/PROFTOT99T)****”*<br><br>**PROF99T:**<br>*People working in a professional occupation in smallest geography (1 Agriculteurs exploitants, 2 Artisans, commerçants, chefs d'entr., 3 Cadres, professions intel. Supérieures, 4 Professions intermédiaires)*<br><br>**PROFTOT99T:**

		**Data source:** *INSEE (1999; 2006-2013)*<br><br>***<br>**Unit of Analysis***: IRIS*	*(bracket) by diploma (1999)*<br><br>***<br>**Data source:** *INSEE (1999; 2006-2013)*<br><br>***<br>**Unit of Analysis***: IRIS*			***<br>**Data source:** *INSEE (1999; 2006-2013)*<br><br>***<br>**Unit of Analysis***: IRIS*	*Occupation total denominator; Total population 15 years +*<br><br>***<br>**Data source:** *INSEE (1999; 2006-2013)*<br><br>***<br>**Unit of Analysis***: IRIS*
**Bristol** (United Kingdom) **Sheffield** (United Kingdom) **Edinburgh** (United Kingdom)	2001	**NVULN01T**<br>*"Percentage of Non-vulnerable community (**NVULN01T/VULTOT01T**)"*<br><br>**NVULN01T**:<br>*Aggregate of White British, White Scottish and Other White*<br><br>**VULTOT01T**:<br>*Total answering the vulnerability question; total Race/Ethicity surveyed*<br><br>***<br>**Data source:** *UK Census (1991,2001, 2011)*<br><br>***<br>**Unit of Analysis***: (**Bristol and Sheffield**) Output Area (2001, 2011), (**Edinburgh**) Output Area*	**UNI01T**<br>*"Percentage of people with a university degree or higher (**UNI01T/EDUTOT01T**)*<br><br>**UNI01T**:<br>Qualified (Aggregate of Level a (higher degree), Level b (degree), and Level c (diploma etc.)<br><br>**EDUTOT01T**:<br>Total Education defined by the "Qualified manpower" 10% Sample, Total Persons aged 18 or over<br><br>***<br>**Data source:** *UK Census (1991,2001, 2011)*<br><br>***<br>**Unit of Analysis***: (**Bristol and Sheffield**) Output Area (2001, 2011), (**Edinburgh**) Output Area*	**N/A**	**N/A**	**PC_SHOU01T**<br>*"Percentage of residents <u>not</u> below poverty level (**SHOU01T/HHTOT01T**)"*<br><br>**SHOUT01T:**<br>*Total number of social housing residents (Social Rent (local council, housing association, and other social rent))*<br><br>**HHTOT01T:**<br>*Total number of households surveyed on "Occupancy and Tender"*<br><br>***<br>**Data source:** *UK Census (1991,2001, 2011)*<br><br>***<br>**Unit of Analysis***: (**Bristol and Sheffield**) Output Area (2001, 2011), (**Edinburgh**) Output Area*	**PC_PROF01T**<br>*"Percentage of residents with professional occupation (**PROF01T/PROFTOT01T**)"*<br><br>**PROF01T:**<br>*People working in professional occupation in smallest geography; Aggregate of (1)Managers & Senior Officials + (2)Professional occupations; number of people*<br><br>**PROFTOT01T:**<br>*Occupation total denominator; "Social Class of Households" 10% sample, total persons*<br><br>***<br>**Data source:** *UK Census (1991,2001, 2011)*<br><br>***<br>**Unit of Analysis***: (**Bristol and Sheffield**) Output Area (2001, 2011), (**Edinburgh**) Output Area*
**Amsterdam**(The Netherlands)	2006/2007	**PC_NVULN07W**<br>*"Percentage of people with migration backgorund (i.e. allochtone)(**NVULN07W/VULTOT07W**)*<br><br>**NVULN07W**<br>*Dutch Autochthone population, which applies only if the person and both their parents are born in the Netherlands.*	**PC_UNI06W**<br>*"Percentage of people with higher education(**UNI06W/EDUTOT06W**)*<br><br>**UNI06W:**<br>*2001 and 2006: People over 15 with high education, 2010-2014:*	**AVINC07W**<br>*Average income*<br><br>***<br>**Data source:** *Municipal Research, Information and Statistics Office (1997-2017), Dutch national Statistics Netherlands (1997-2007, 2001, 2006, 2010),*<br><br>***	**AVRENT07W**<br>*Average rent paid*<br><br>***<br>**Data source:** *Municipal Research, Information and Statistics Office (1997-2017), Dutch national Statistics Netherlands (1997-2007, 2001, 2006, 2010),*	**N/A**	**N/A**

		**VULTOT07W**<br>*Total number of people per wijk (neighborhood)*<br><br>***<br>**Data source:** *Municipal Research, Information and Statistics Office (1997-2017), Dutch national Statistics Netherlands (1997-2007, 2001, 2006, 2010),*<br><br>***<br>**Unit of Analysis***: Wijk (neighborhood)*	*People 15-75 with high education*<br><br>**EDUTOT06W:**<br>*Total people for education variables*<br><br>***<br>**Data source:** *Municipal Research, Information and Statistics Office (1997-2017), Dutch national Statistics Netherlands (1997-2007, 2001, 2006, 2010),*<br><br>***<br>**Unit of Analysis***: Wijk (neighborhood)*	**Unit of Analysis***: Wijk (neighborhood*	***<br>**Unit of Analysis***: Wijk (neighborhood*		
**Barcelona**(Spain)	2000/2001	**PC_NVULN01T**<br>"*Percentage of non-vulnerable residents per nationality* ***(GNIMM01T/VULTOT01T)***"<br><br>**GNIMM01T:**<br>*NON-vulnerable countries per nationality, EU27, USA, Canada, Norway, Luxembourg, Andorra, island, Litchestein*<br><br>**VULTOT01T:**<br>*total population (GSIMM01T+GNIMM01T)*<br><br>***<br>**Data source:** *For age, profession, education, vulnerability: Instituto Nacional de Estadística (1991, 2001, 2011) and register of inhabitants (1996 or 1997, 2006, 2016)*<br><br>***<br>**Unit of Analysis***: Census tract, Small Research Area or Neighborhood depending on year and variable*	**PC_UNI01T**<br>"*Percentage of people with university degree or higher* ***(UNI01T/EDUTOT01T)***"<br><br>**UNI01T**:<br>*University studies OR CFGS higher degree*<br><br>**EDUTOT01T:**<br>*total population for education variable*<br><br>***<br>**Data source:** *For age, profession, education, vulnerability: Instituto Nacional de Estadística (1991, 2001, 2011) and register of inhabitants (1996 or 1997, 2006, 2016)*<br><br>***<br>**Unit of Analysis***: Census tract, Small Research Area or Neighborhood depending on year and variable*	**INCOME00T**<br><br>**RFDVALUE00R**: *Level of people considered high income*<br><br>***<br>**Data source:** *For age, profession, education, vulnerability: Instituto Nacional de Estadística (1991, 2001, 2011) and register of inhabitants (1996 or 1997, 2006, 2016)*<br><br>***<br>**Unit of Analysis***: Census tract, Small Research Area or Neighborhood depending on year and variable*	**RENT01T**<br><br>**RENTSM01ZEG:**<br>*Rent price per month in Euro/m2 for second hand renters.*<br><br>***<br>**Data source:** *For age, profession, education, vulnerability: Instituto Nacional de Estadística (1991, 2001, 2011) and register of inhabitants (1996 or 1997, 2006, 2016)*<br><br>***<br>**Unit of Analysis***: Census tract, Small Research Area or Neighborhood depending on year and variable*	**N/A**	**N/A**

**Copenhagen**(Denmark)	N/A	**N/A**	**N/A**	**N/A**	**N/A**	**N/A**	**N/A**
**Dublin** (Ireland)	2006	**PC_NVULN06E**<br>*"Percentage of non-vulnerable community (**NVULN06E/VULTOT06E**)"*<br><br>**NVULN06E:**<br>*Non-vulnerable community; White Irish*<br><br>**VULTOT06E:**<br>*Total number of people answering the vulnerability question; Total Persons surveyed 'usually resident population by ethnic or cultural background'*<br><br>***<br>**Data source:** *Ireland Census (1996, 2002, 2006, 2011, 2016)*<br><br>***<br>**Unit of Analysis***: Electoral Divisions (called SAPS in 1996)*	**PC_UNI06E**<br>*"Percentage of residents with a university degree or higher (**UNI06E/EDUTOT06E**)"*<br><br>**UNI06E:**<br>*People with a university degree or higher; aggregate of various degrees and qualifications*<br><br>**EDUTOT06E:**<br>*Population over 15yo by highest level of education completed*<br><br>***<br>**Data source:** *Ireland Census (1996, 2002, 2006, 2011, 2016)*<br><br>***<br>**Unit of Analysis***: Electoral Divisions (called SAPS in 1996)*	**N/A**	**N/A**	**PC_NPOV06E**<br>*"Percentage of households that live in social housing (**SHOU06E/HHTOT06E)"***<br><br>**SHOU06E:**<br>*Number of households that live in social housing*<br><br>**HHTOT06E:**<br>*Total number of households; Total households surveyed on 'Permanent private households by type of occupancy'. All owners, all renters, and all 'living rent-free'/'occupied free of rent'/'not stated'*<br><br>***<br>**Data source:** *Ireland Census (1996, 2002, 2006, 2011, 2016)*<br><br>***<br>**Unit of Analysis***: Electoral Divisions (called SAPS in 1996)*	**PC_PROF06E**<br>***"****Percentage of people working in a professional occupation (**PROF06E/PROFTOT06E**)"*<br><br>**PROF06E:**<br>*People working in a professional occupation; Aggregate of (1) Employers & Managers and (2) Higher professional*<br><br>**PROFTOT06E:**<br>*Total occupation; Total persons; Occupation total denominator*<br><br>***<br>**Data source:** *Ireland Census (1996, 2002, 2006, 2011, 2016)*<br><br>***<br>**Unit of Analysis***: Electoral Divisions (called SAPS in 1996)*
**Valencia**(Spain)	2001	**PC_NVULN01**<br>*"Percentage of non-vulnerable community (**NVULN01T/VULTOT01T**)*<br><br>**NVULN01T**:<br>*Countries of birth: Countries in Africa, Philippines, Peru, Pakistan, Bolivia, Ecuador,*	**PC_UNI01**<br>*"Percentage of people with a university degree or higher (**UNI01T/EDUTOT01T**)*<br><br>**UNI01T:**	**N/A**	**N/A**	**N/A**	**N/A**

		Colombia, or the Dominican Republic<br><br>**VULTOT01T**:<br>*Total population*<br><br>***<br>**Data source:** *For age, profession, education, vulnerability: Instituto Nacional de Estadística censuses (1991, 2001, 2011) and register of inhabitants (1996, 2006, 2016).*<br><br>***<br>**Unit of Analysis***: Census tract or neighborhood depending on year and variable*	*People with a university degree or higher in smallest geography*<br><br>**EDUTOT01T:**<br>*Total population*<br><br>**<br>**Data source:** *For age, profession, education, vulnerability: Instituto Nacional de Estadística censuses (1991, 2001, 2011) and register of inhabitants (1996, 2006, 2016).*<br><br>***<br>**Unit of Analysis***: Census tract or neighborhood depending on year and variable*				
**Vienna** (Austria)	2001	**N/A**	**PC_UNI01T**<br>*"Percentage of residents with university degree (**UNI01T/EDUTOT01T**)"*<br><br>**UNI01T:**<br>*All people with university degree*<br><br>**EDUTOT01T:**<br>*Total number of all categories (total population)*<br><br>***<br>**Data source:** *For age, education, profession, socioeconomic status, and vulnerability: City of Vienna Austrian Public Data Portal (1991, 2001, 2014 or 2015 or 2016)*<br><br>***<br>**Unit of Analysis***: Zählgebiet (Sub-Districts)*	**PC_HISES01T**<br>*"Percentage of residents with high income (**HISES01T/SESTOT01T**)"*<br><br>**HISES01T:**<br>*residents in Sector groups: D,E,J,K,L,S,M,N,O,P,Q,R,U (high socioeconomic status)*<br><br>**SESTOT01T:**<br>*Total number of call categories (total population)*<br><br>***<br>**Data source:** *For age, education, profession, socioeconomic status, and vulnerability: City of Vienna Austrian Public Data Portal (1991, 2001, 2014 or 2015 or 2016)*<br><br>***<br>**Unit of Analysis***: Zählgebiet (Sub-Districts)*	**N/A**	**N/A**	**PC_PROF01T**<br>*"Percentage of residents with professional occupation (**PROF01T/PROFTOT01T**)*<br><br>**PROF01T:**<br>*People working in professional education*<br><br>**PROFTOT01T:**<br>*Total number of all categories (total population)*<br><br>***<br>**Data source:** *For age, education, profession, socioeconomic status, and vulnerability: City of Vienna Austrian Public Data Portal (1991, 2001, 2014 or 2015 or 2016)*<br><br>***<br>**Unit of Analysis***: Zählgebiet (Sub-Districts)*

Table S2 – APPROXIMATELY YEAR 2010

City	Year represented	Vulnerability	Education	Income	Real estate information	Poverty	Professionals
**Atlanta**(Georgia, US)<br>**Austin** (Texas, US)<br>**Baltimore** (Maryland, US)<br>**Boston** (Massachusetts, US)<br>**Cleveland**(Ohio, US)<br>**Denver** (Colorado, US)<br>**Detroit**(Michigan, US)<br>**Louisville**(Kentucky, US)<br>**Milwaukee**(Wisconsin, US)<br>**Philadelphia**(Pennsylvania, US)<br>**Portland**(Oregon, US)<br>**San Francisco**(California, US)<br>**Seattle**(Washington, US)<br>**Washington DC** (District of Columbia, US)	2010	**PC_NVULN10T**<br>*"Percentage of non-Hispanic White residents (**NVULN10T/VULTOT10T**)"*<br><br>**NVULN10T:**<br>*Non-Hispanic white only*<br><br>**VULTOT10T:**<br>*Total number of people for race/ethnicity variable*<br><br>***<br>**Data source:** *US Census (1990-2010), American Community Survey (2006-2010 and 2012-2016), Data standardized to 2010 census tracts purchased from GeoLytics (Neighborhood Change database)*<br><br>***<br>**Unit of Analysis***: Census Tract*	**PC_UNI10T**<br>*"Percentage of residents with university degree or higher (**UNI10T/EDUTOT10T**)"*<br><br>**UNI10T:**<br>*people with university degree or higher*<br><br>**EDUTOT10T:**<br>*Total number of people for education variables*<br><br>***<br>**Data source:** *US Census (1990-2010), American Community Survey (2006-2010 and 2012-2016), Data standardized to 2010 census tracts purchased from GeoLytics (Neighborhood Change database)*<br><br>***<br>**Unit of Analysis***: Census Tract*	**PC_HISES10T***"Percentage of residents with high income (**HISES10T/SESTOT10T**)"*<br><br>**HISES10T:**<br>*Household income categories, adjusted for each city such that low SES = those in categories above 125% of the median for the city for the year*<br><br>**SESTOT10T**:<br>*Used aggregate of all categories rather than provided denominator due to error because of the standardization of the census tracts to 2010 areas according to suggestions from Geolytics*<br><br>***<br>**Data source:** *US Census (1990-2010), American Community Survey (2006-2010 and 2012-2016), Data standardized to 2010 census tracts purchased from GeoLytics (Neighborhood Change database)*<br><br>***<br>**Unit of Analysis***: Census Tract*	**PC_HRENT10T***"Percentage of households paying above median value in rent (**HRENT10T/RENT10T**)"*<br><br>***HRENT10T:***<br>*Rental households paying above the median value in rent, adjusted for each city and year to the median rent for the city for the year*<br><br>**RENT10T:**<br>*Used aggregate of all rent categories rather than provided denominator due to error because of the standardization of the census tracts to 2010 areas according to suggestions from Geolytics*<br><br>***<br>**Data source:** *US Census (1990-2010), American Community Survey (2006-2010 and 2012-2016), Data standardized to 2010 census tracts purchased from GeoLytics (Neighborhood Change database)*<br><br>***<br>**Unit of Analysis***: Census Tract*	**PC_NPOV10T**<br>*"Percentage of residents not below poverty level (**POV10T/POVTOT10T**)"*<br><br>**POV10T**:<br>*Number of people determined to be below poverty level*<br><br>**POVTOT10T**:<br>*Total number for which poverty is determined*<br><br>***<br>**Data source:** *US Census (1990-2010), American Community Survey (2006-2010 and 2012-2016), Data standardized to 2010 census tracts purchased from GeoLytics (Neighborhood Change database)*<br><br>***<br>**Unit of Analysis***: Census Tract*	**PC_PROF10T**<br>*"Percentage of residents with professional occupation (**PROF10T/PROFTOT10T**)"*<br><br>**PROF00T:**<br>*Aggregate of two categories: 1) Professional, scientific, and management, and administrative and waste management services: - Professional, scientific, and technical services and 2) Professional, scientific, and management, and administrative and waste management services: - Management of companies and enterprises*<br><br>**PROFTOT10T:**<br>*Total number of people asked about occupation*<br><br>***<br>**Data source:** *US Census (1990-2010), American Community Survey (2006-2010 and 2012-2016), Data standardized to 2010 census tracts purchased from GeoLytics (Neighborhood Change database)*<br><br>***<br>**Unit of Analysis***: Census Tract*
**Calgary**(Canada)<br>**Montreal**(Canada)<br>**Vancouver**(Canada)	2011	**PCNVULN11T**<br>*"Percentage of Non-vulnerable community (**VISMIN11T/VISMINT0T11T**")*<br><br>**VISMIN11T:**	**PCUNI11T**<br>*"Percentage of residents with university degree or higher (**UNI11T/EDUTOT11T**)"*<br><br>**UNI11T:**	**PC_HISES11T**<br>*"Percentage of residents with high income (**HISES11T/SESTOT11T**)"*<br><br>**HISES11T:**	**PC_HRENT11T**<br>*"Percentage of households paying above median value in rent(**HRENT11T/RENT11T**)"*	**N/A**	**PC_PROF11T**<br>*"Percentage of residents with professional occupation (**PROF11T/PROFTOT11T**)*<br><br>**PROF11T:**

		Total population in private households by visible minority (Black, Arab, S. Asian and multiple visible minorities).<br><br>**VISMINT0T11T:**<br>*Total population by visible minority*<br><br>***<br>**Data source:** *Canadian Census (1991, 1996, 2001, 2006, 2011, 2016)*<br><br>***<br>**Unit of Analysis***: Enumeration areas (1991, 1996); Dissemination areas (post 1996)*	*Population 20+ with university degree or higher*<br><br>**EDUTOT11T:**<br>*Total number of people for education variables per EA or DA*<br><br>***<br>**Data source:** *Canadian Census (1991, 1996, 2001, 2006, 2011, 2016)*<br><br>***<br>**Unit of Analysis***: Enumeration areas (1991, 1996); Dissemination areas (post 1996)*	*High SES/Total for this variable (125% above city median household income)*<br><br>**SES11T:**<br>*Total SES (Household income - all private households)*<br><br>***<br>**Data source:** *Canadian Census (1991, 1996, 2001, 2006, 2011, 2016)*<br><br>***<br>**Unit of Analysis***: Enumeration areas (1991, 1996); Dissemination areas (post 1996)*	**HRENT11T**<br>*Rental households paying above the median value in rent, adjusted for each city and year to the median rent for the city for the year*<br><br>**RENT11T:**<br>*Total number of renters*<br><br>***<br>**Data source:** *Canadian Census (1991, 1996, 2001, 2006, 2011, 2016)*<br><br>***<br>**Unit of Analysis***: Enumeration areas (1991, 1996); Dissemination areas (post 1996)*		*People working a professional occupation; 20+ categories*<br><br>**PROFTOT11T:**<br>*Total occupation/occupation total denominator*<br><br>***<br>**Data source:** *Canadian Census (1991, 1996, 2001, 2006, 2011, 2016)*<br><br>***<br>**Unit of Analysis***: Enumeration areas (1991, 1996); Dissemination areas (post 1996)*
**Lyon** (France)<br>**Nantes** (France)	2013	**PC_NVULN13**<br>*"Percentage of non-vulnerable residents (1-**VULN13T/VULTOT13T**)"*<br><br>**VULN13T:**<br>*Vulnerable community (specify)- smallest geography /PLACE OF BIRTH - Foreign (1999); Number of immigrants (2006-2013)*<br><br>**VULTOT13T:**<br>Total population, smallest geography<br><br>***<br>**Data source:** *INSEE (1999; 2006-2013)*<br><br>***<br>**Unit of Analysis***: IRIS*	**PC_UNI13**<br>*"Percentage of residents with university degree or higher (**UNI13T/EDUTOT13T**)"*<br><br>**UNI13T:**<br>*People with a university degree or higher / Senior Degrees*<br><br>**EDUTOT13T:**<br>*Education total, smallest geography / Population 15 years and over by sex, age (bracket) by diploma (1999)*<br><br>***<br>**Data source:** *INSEE (1999; 2006-2013)*<br><br>***<br>**Unit of Analysis***: IRIS*	**MEDINC13T:**<br>*Median income, smallest geography*<br><br>***<br>**Data source:** *INSEE (1999; 2006-2013)*<br><br>***<br>**Unit of Analysis***: IRIS*	**N/A**	**PC_NPOV13**<br>*"Percentage of social housing primary residences not below poverty level (**1-HLMV13T/HHTOT13T**)"*<br><br>**HLMV13T:**<br>*Number of social housing , primary residences rented empty - an empty rented accommodation HLM*<br><br>**HHTOT13T:**<br>*Total number of primary residences (denominator)/ Number of main residences*<br><br>***<br>**Data source:** *INSEE (1999; 2006-2013)*<br><br>***<br>**Unit of Analysis***: IRIS*	**PC_PROF13**<br>*"Percentage of residents with professional occupation (**PROF13T/PROFTOT13T**)"*<br><br>**PROF13T:**<br>*People working in a professional occupation in smallest geography (1 Agriculteurs exploitants, 2 Artisans, commerçants, chefs d'entr., 3 Cadres, professions intel. Supérieures, 4 Professions intermédiaires)*<br><br>**PROFTOT13T:**<br>*Occupation total denominator; Total population 15 years +*<br><br>***<br>**Data source:** *INSEE (1999; 2006-2013)*<br><br>***<br>**Unit of Analysis***: IRIS*
**Bristol** (United Kingdom)	2011	**NVULN11T**	**UNI01T**<br>*"Percentage of people with a university degree or*	**N/A**	**N/A**	**PC_SHOU11T**	**PC_PROF11T**

**Sheffield** (United Kingdom)<br>**Edinburgh** (United Kingdom)		*"Percentage of Non-vulnerable community (**NVULN11/VULTOT11**)"*<br><br>**NVULN11T**:<br>*Aggregate of White British, White Scottish and Other White*<br><br>**VULTOT11T**:<br>*Total answering the vulnerability question; total Race/Ethicity surveyed*<br><br>***<br>**Data source:** *UK Census (1991,2001, 2011)*<br><br>***<br>**Unit of Analysis**: *(**Bristol and Sheffield**) Output Area (2001, 2011), (**Edinburgh**) Output Area*	*higher (**UNI11/EDUTOT11**)*<br><br>**UNI11T**:<br>Qualified (Aggregate of Level a (higher degree), Level b (degree), and Level c (diploma etc.)<br><br>**EDUTOT11T**:<br>Total Education defined by the "Qualified manpower" 10% Sample, Total Persons aged 18 or over<br><br>***<br>**Data source:** *UK Census (1991,2001, 2011)*<br><br>***<br>**Unit of Analysis**: *(**Bristol and Sheffield**) Output Area (2001, 2011), (**Edinburgh**) Output Area*			*"Percentage of residents <u>not</u> below poverty level (**SHOU11T/HHTOT11T**)"*<br><br>**SHOUT11T:**<br>*Total number of social housing residents (Social Rent (local council, housing association, and other social rent))*<br><br>**HHTOT11T:**<br>*Total number of households surveyed on "Occupancy and Tender"*<br><br>***<br>**Data source:** *UK Census (1991,2001, 2011)*<br><br>***<br>**Unit of Analysis**: *(**Bristol and Sheffield**) Output Area (2001, 2011), (**Edinburgh**) Output Area*	*"Percentage of residents with professional occupation (**PROF11T/PROFTOT11T**)"*<br><br>**PROF11T:**<br>*People working in professional occupation in smallest geography; Aggregate of (1)Managers & Senior Officials + (2)Professional occupations; number of people*<br><br>**PROFTOT11T:**<br>*Occupation total denominator; "Social Class of Households" 10% sample, total persons*<br><br>***<br>**Data source:** *UK Census (1991,2001, 2011)*<br><br>***<br>**Unit of Analysis**: *(**Bristol and Sheffield**) Output Area (2001, 2011), (**Edinburgh**) Output Area*
**Amsterdam**(The Netherlands)	2009/2010	**PC_NVULN10W**<br>*"Percentage of people with migration backgorund (i.e. allochtone)(**NVULN10W/VULTOT10W**)*<br><br>**NVULN10W**<br>*Dutch Autochthone population, which applies only if the person and both their parents are born in the Netherlands.*<br><br>**VULTOT10W**<br>*Total number of people per wijk (neighborhood)*<br><br>***<br>**Data source:** *Municipal Research, Information and Statistics Office (1997-2017), Dutch national Statistics Netherlands (1997-2007, 2001, 2006, 2010),*	**PC_UNI10W**<br>*"Percentage of people with higher education(**UNI10W/EDUTOT10W**)*<br><br>**UNI10W:**<br>*2001 and 2006: People over 15 with high education, 2010-2014: People 15-75 with high education*<br><br>**EDUTOT10W:**<br>*Total people for education variables*<br><br>***<br>**Data source:** *Municipal Research, Information and Statistics Office (1997-2017), Dutch*	**AVINC10W**<br>*Average income*<br><br>***<br>**Data source:** *Municipal Research, Information and Statistics Office (1997-2017), Dutch national Statistics Netherlands (1997-2007, 2001, 2006, 2010),*<br><br>***<br>**Unit of Analysis**: *Wijk (neighborhood*	**AVRENT09W**<br>*Average rent paid*<br><br>***<br>**Data source:** *Municipal Research, Information and Statistics Office (1997-2017), Dutch national Statistics Netherlands (1997-2007, 2001, 2006, 2010),*<br><br>***<br>**Unit of Analysis**: *Wijk (neighborhood*	**N/A**	**N/A**

		<br>**Unit of Analysis: Wijk (neighborhood)*	*national Statistics Netherlands (1997-2007, 2001, 2006, 2010),*<br><br>***<br>**Unit of Analysis***: Wijk (neighborhood)*				
**Barcelona**(Spain)	2011	**PC_NVULN11T**<br>"*Percentage of non-vulnerable residents per nationality* ***(GNIMM11T/VULTOT11T)"***<br><br>**GNIMM11T:**<br>*NON-vulnerable countries per nationality, EU27, USA, Canada, Norway, Luxembourg, Andorra, island, Litchestein*<br><br>**VULTOT11T:**<br>*total population (GSIMM01T+GNIMM01T)*<br><br>***<br>**Data source:** *For age, profession, education, vulnerability: Instituto Nacional de Estadística (1991, 2001, 2011) and register of inhabitants (1996 or 1997, 2006, 2016)*<br><br>***<br>**Unit of Analysis***: Census tract, Small Research Area or Neighborhood depending on year and variable*	**PC_UNI11T**<br>*"Percentage of people with university degree or higher* ***(UNI11T/EDUTOT11T)"***<br><br>**UNI11T**:<br>*University studies OR CFGS higher degree*<br><br>**EDUTOT11T:**<br>*total population for education variable*<br><br>***<br>**Data source:** *For age, profession, education, vulnerability: Instituto Nacional de Estadística (1991, 2001, 2011) and register of inhabitants (1996 or 1997, 2006, 2016)*<br><br>***<br>**Unit of Analysis***: Census tract, Small Research Area or Neighborhood depending on year and variable*	**INCOME11T**<br><br>**RFDVALUE11R**: *Level of people considered high income*<br><br>***<br>**Data source:** *For age, profession, education, vulnerability: Instituto Nacional de Estadística (1991, 2001, 2011) and register of inhabitants (1996 or 1997, 2006, 2016)*<br><br>***<br>**Unit of Analysis***: Census tract, Small Research Area or Neighborhood depending on year and variable*	**RENT11T**<br><br>**RENTSM11ZEG:**<br>*Rent price per month in Euro/m2 for second hand renters.*<br><br>***<br>**Data source:** *For age, profession, education, vulnerability: Instituto Nacional de Estadística (1991, 2001, 2011) and register of inhabitants (1996 or 1997, 2006, 2016)*<br><br>***<br>**Unit of Analysis***: Census tract, Small Research Area or Neighborhood depending on year and variable*	**N/A**	**N/A**
**Copenhagen**(Denmark)	2009	**PC_NVUL09R**<br>*"Percentage of non-vulnerable community* ***(NVUL09R/VULNTOT09R)"***	**PC_UNI09R**<br>*"Percentage of residents with a university degree* ***(UNI09R/EDUTOT09R)"***	**PC_HISES09R**<br>**"***Percentage of residents with high-income* **(HISES09R/SESTOT09R)** *"*	**N/A**	**PC_PUBHO09R**<br>*"Percentage of residents not below poverty level* ***(PUB09R/TOTHH09R)***	**N/A**

		**NVUL09R:**<br>*Population with origins in Denmark or other Western countries*<br><br>**VULTOT09R:**<br>*Total of people answering vulnerability question*<br><br>***<br>**Data source:** *Danish Statistical Office (yearly from 1990 to 2016)*<br><br>***<br>**Unit of Analysis***: Roder*	**UNI09R:**<br>*Population with a university degree or higher*<br><br>**EDUTOT09R:**<br>*Total education*<br><br>***<br>**Data source:** *Danish Statistical Office (yearly from 1990 to 2016)*<br><br>***<br>**Unit of Analysis***: Roder*	**HISES09R:**<br>*High income*<br><br>**SESTOT09R:**<br>*Aggregate of all high income*<br><br>***<br>**Data source:** *Danish Statistical Office (yearly from 1990 to 2016)*<br><br>***<br>**Unit of Analysis***: Roder*		**PUB09R:**<br>*Those living in public housing*<br><br>**TOTHH09R:**<br>*Total household (denominator for housing)*<br><br>***<br>**Data source:** *Danish Statistical Office (yearly from 1990 to 2016)*<br><br>***<br>**Unit of Analysis***: Roder*	
**Dublin** (Ireland)	2011	**PC_NVULN11E**<br>*"Percentage of non-vulnerable community* ***(NVULN11E/VULTOT11E)****"*<br><br>**NVULN11E:**<br>*Non-vulnerable community; White Irish*<br><br>**VULTOT11E:**<br>*Total number of people answering the vulnerability question; Total Persons surveyed 'usually resident population by ethnic or cultural background'*<br><br>***<br>**Data source:** *Ireland Census (1996, 2002, 2006, 2011, 2016)*<br><br>***<br>**Unit of Analysis***: Electoral Divisions (called SAPS in 1996)*	**PC_UNI11E**<br>*"Percentage of residents with a university degree or higher* *(****UNI11E/EDUTOT11E****)"*<br><br>**UNI11E:**<br>*People with a university degree or higher; aggregate of various degrees and qualifications*<br><br>**EDUTOT11E:**<br>*Population over 15yo by highest level of education completed*<br><br>***<br>**Data source:** *Ireland Census (1996, 2002, 2006, 2011, 2016)*<br><br>***<br>**Unit of Analysis***: Electoral Divisions (called SAPS in 1996)*	**N/A**	**N/A**	**PC_NPOV11E**<br>*"Percentage of households that live in social housing* ***(SHOU11E/HHTOT11E)"***<br><br>**SHOU11E:**<br>*Number of households that live in social housing*<br><br>**HHTOT11E:**<br>*Total number of households; Total households surveyed on 'Permanent private households by type of occupancy'. All owners, all renters, and all 'living rent-free'/'occupied free of rent'/'not stated'*<br><br>***<br>**Data source:** *Ireland Census (1996, 2002, 2006, 2011, 2016)*<br><br>***<br>**Unit of Analysis***: Electoral Divisions (called SAPS in 1996)*	**PC_PROF11E**<br>***"****Percentage of people working in a professional occupation* ***(PROF11E/PROFTOT11E)****"*<br><br>**PROF11E:**<br>*People working in a professional occupation; Aggregate of (1) Employers & Managers and (2) Higher professional*<br><br>**PROFTOT11E:**<br>*Total occupation; Total persons; Occupation total denominator*<br><br>***<br>**Data source:** *Ireland Census (1996, 2002, 2006, 2011, 2016)*<br><br>***<br>**Unit of Analysis***: Electoral Divisions (called SAPS in 1996)*

**Valencia**(Spain)	2011	**PC_NVULN11T**<br>*"Percentage of non-vulnerable community (**NVULN11T/VULTOT01T**)*<br><br>**NVULN11T**:<br>*Countries of birth: Countries in Africa, Philippines, Peru, Pakistan, Bolivia, Ecuador, Colombia, or the Dominican Republic*<br><br>**VULTOT11T**:<br>*Total population*<br><br>***<br>**Data source:** *For age, profession, education, vulnerability: Instituto Nacional de Estadística censuses (1991, 2001, 2011) and register of inhabitants (1996, 2006, 2016).*<br><br>***<br>**Unit of Analysis***: Census tract or neighborhood depending on year and variable*	**PC_UNI11T**<br>*"Percentage of people with a university degree or higher (**UNI11T/EDUTOT11T**)*<br><br>**UNI11T:**<br>*People with a university degree or higher in smallest geography*<br><br>**EDUTOT11T:**<br>*Total population*<br><br>**<br>**Data source:** *For age, profession, education, vulnerability: Instituto Nacional de Estadística censuses (1991, 2001, 2011) and register of inhabitants (1996, 2006, 2016).*<br><br>***<br>**Unit of Analysis***: Census tract or neighborhood depending on year and variable*	**N/A**	**N/A**	**N/A**	**N/A**
**Vienna** (Austria)	N/A	**N/A**	**N/A**	**N/A**	**N/A**	**N/A**	**N/A**

Table S3 – APPROXIMATELY YEAR 2016

City	Year represented	Vulnerability	Education	Income	Real estate information	Poverty	Professionals
**Atlanta**(Georgia, US)<br>**Austin** (Texas, US)<br>**Baltimore** (Maryland, US)	2016	**PC_NVULN16T**<br>*"Percentage of non-Hispanic White residents (**NVULN16T/VULTOT16T**)"*<br><br>**NVULN16T:**<br>*Non-Hispanic white only*	**PC_UNI16T**<br>*"Percentage of residents with university degree or higher (**UNI16T/EDUTOT16T**)"*<br><br>**UNI16T:**	**PC_HISES16T***"Percentage of residents with high income (**HISES16T/SESTOT16T**)"*<br><br>**HISES16T:**<br>*Household income categories, adjusted for each*	**PC_HRENT16T***"Percent age of households paying above median value in rent (**HRENT16T/RENT16T**)"*<br><br>***HRENT16T:***	**PC_NPOV16T**<br>*"Percentage of residents not below poverty level (**POV10T/POVTOT10T**)"*<br><br>**POV16T**:	**PC_PROF16T**<br>*"Percentage of residents with professional occupation (**PROF16T/PROFTOT16T**)"*<br><br>**PROF16T:**

**Boston** (Massachusetts, US)<br>**Cleveland**(Ohio, US)<br>**Denver** (Colorado, US)<br>**Detroit**(Michigan, US)<br>**Louisville**(Kentucky, US)<br>**Milwaukee**(Wisconsin, US)<br>**Philadelphia**(Pennsylvania, US)<br>**Portland**(Oregon, US)<br>**San Francisco**(California, US)<br>**Seattle**(Washington, US)<br>**Washington DC** (District of Columbia, US)		**VULTOT16T:**<br>*Total number of people for race/ethnicity variable*<br><br>***<br>**Data source:** *US Census (1990-2010), American Community Survey (2006-2010 and 2012-2016), Data standardized to 2010 census tracts purchased from GeoLytics (Neighborhood Change database)*<br><br>***<br>**Unit of Analysis***: Census Tract*	*people with university degree or higher*<br><br>**EDUTOT16T:**<br>*Total number of people for education variables*<br><br>***<br>**Data source:** *US Census (1990-2010), American Community Survey (2006-2010 and 2012-2016), Data standardized to 2010 census tracts purchased from GeoLytics (Neighborhood Change database)*<br><br>***<br>**Unit of Analysis***: Census Tract*	*city such that low SES = those in categories above 125% of the median for the city for the year*<br><br>**SESTOT16T**:<br>*Used aggregate of all categories rather than provided denominator due to error because of the standardization of the census tracts to 2010 areas according to suggestions from Geolytics*<br><br>***<br>**Data source:** *US Census (1990-2010), American Community Survey (2006-2010 and 2012-2016), Data standardized to 2010 census tracts purchased from GeoLytics (Neighborhood Change database)*<br><br>***<br>**Unit of Analysis***: Census Tract*	*Rental households paying above the median value in rent, adjusted for each city and year to the median rent for the city for the year*<br><br>**RENT16T:**<br>*Used aggregate of all rent categories rather than provided denominator due to error because of the standardization of the census tracts to 2010 areas according to suggestions from Geolytics*<br><br>***<br>**Data source:** *US Census (1990-2010), American Community Survey (2006-2010 and 2012-2016), Data standardized to 2010 census tracts purchased from GeoLytics (Neighborhood Change database)*<br><br>***<br>**Unit of Analysis***: Census Tract*	*Number of people determined to be below poverty level*<br><br>**POVTOT16T**:<br>*Total number for which poverty is determined*<br><br>***<br>**Data source:** *US Census (1990-2010), American Community Survey (2006-2010 and 2012-2016), Data standardized to 2010 census tracts purchased from GeoLytics (Neighborhood Change database)*<br><br>***<br>**Unit of Analysis***: Census Tract*	*Aggregate of two categories: 1) Professional, scientific, and management, and administrative and waste management services: - Professional, scientific, and technical services and 2) Professional, scientific, and management, and administrative and waste management services: - Management of companies and enterprises*<br><br>**PROFTOT16T:**<br>*Total number of people asked about occupation*<br><br>***<br>**Data source:** *US Census (1990-2010), American Community Survey (2006-2010 and 2012-2016), Data standardized to 2010 census tracts purchased from GeoLytics (Neighborhood Change database)*<br><br>***<br>**Unit of Analysis***: Census Tract*
**Calgary**(Canada)<br>**Montreal**(Canada)<br>**Vancouver**(Canada)	2016	**PCNVULN16T**<br>*"Percentage of Non-vulnerable community (**VISMIN16T/VISMINT0T16T"**)*<br><br>**VISMIN16T:**<br>*Total population in private households by visible minority (Black, Arab, S. Asian and multiple visible minorities).*<br><br>**VISMINT0T16T:**<br>*Total population by visible minority*<br><br>***<br>**Data source:** *Canadian Census (1991, 1996, 2001, 2006, 2011, 2016)*	**PCUNI16T**<br>*"Percentage of residents with university degree or higher (**UNI16T/EDUTOT16T**)"*<br><br>**UNI16T:**<br>*Population 20+ with university degree or higher*<br><br>**EDUTOT16T:**<br>*Total number of people for education variables per EA or DA*<br><br>***<br>**Data source:** *Canadian Census (1991, 1996, 2001, 2006, 2011, 2016)*<br><br>***	**PC_HISES16T**<br>*"Percentage of residents with high income (**HISES11T/SESTOT11T**)"*<br><br>**HISES16T:**<br>*High SES/Total for this variable (125% above city median household income)*<br><br>**SES16T:**<br>*Total SES (Household income - all private households)*<br><br>***<br>**Data source:** *Canadian Census (1991, 1996, 2001, 2006, 2011, 2016)*	**PC_HRENT16T**<br>*"Percentage of households paying above median value in rent(**HRENT16T/RENT16T**)"*<br><br>**HRENT16T**<br>*Rental households paying above the median value in rent, adjusted for each city and year to the median rent for the city for the year*<br><br>**RENT16T:**<br>*Total number of renters*<br><br>***<br>**Data source:** *Canadian Census (1991, 1996, 2001, 2006, 2011, 2016)*	**N/A**	**PC_PROF16T**<br>*"Percentage of residents with professional occupation (**PROF11T/PROFTOT11T)***<br><br>**PROF16T:**<br>*People working a professional occupation; 20+ categories*<br><br>**PROFTOT16T:**<br>*Total occupation/occupation total denominator*<br><br>***<br>**Data source:** *Canadian Census (1991, 1996, 2001, 2006, 2011, 2016)*<br><br>***<br>**Unit of Analysis***: Enumeration areas (1991,*

		*** **Unit of Analysis***: Enumeration areas (1991, 1996); Dissemination areas (post 1996)*	**Unit of Analysis***: Enumeration areas (1991, 1996); Dissemination areas (post 1996)*	*** **Unit of Analysis***: Enumeration areas (1991, 1996); Dissemination areas (post 1996)*	*** **Unit of Analysis***: Enumeration areas (1991, 1996); Dissemination areas (post 1996)*		*1996); Dissemination areas (post 1996)*
**Lyon** (France) **Nantes** (France)	N/A	**N/A**	**N/A**	**N/A**	**N/A**	**N/A**	**N/A**
**Bristol** (United Kingdom) **Sheffield** (United Kingdom) **Edinburgh** (United Kingdom)	N/A	**N/A**	**N/A**	**N/A**	**N/A**	**N/A**	**N/A**
**Amsterdam**(The Netherlands)	2013/2014	**PC_NVULN14W** *"Percentage of people with migration backgorund (i.e. allochtone)****(NVULN14W/VULTOT14W)*** <br>**NVULN14W** *Dutch Autochthone population, which applies only if the person and both their parents are born in the Netherlands.* <br>**VULTOT14W** *Total number of people per wijk (neighborhood)* <br>*** **Data source:** *Municipal Research, Information and Statistics Office (1997-2017), Dutch national Statistics Netherlands (1997-2007, 2001, 2006, 2010),* <br>*** **Unit of Analysis***: Wijk (neighborhood)*	**PC_UNI14W** *"Percentage of people with higher education****(UNI14W/EDUTOT14W)*** <br>**UNI14W:** *2001 and 2006: People over 15 with high education, 2010-2014: People 15-75 with high education* <br>**EDUTOT14W:** *Total people for education variables* <br>*** **Data source:** *Municipal Research, Information and Statistics Office (1997-2017), Dutch national Statistics Netherlands (1997-2007, 2001, 2006, 2010),* <br>*** **Unit of Analysis***: Wijk (neighborhood)*	**AVINC14W** *Average income* <br>*** **Data source:** *Municipal Research, Information and Statistics Office (1997-2017), Dutch national Statistics Netherlands (1997-2007, 2001, 2006, 2010),* <br>*** **Unit of Analysis***: Wijk (neighborhood*	**AVRENT13W** *Average rent paid* <br>*** **Data source:** *Municipal Research, Information and Statistics Office (1997-2017), Dutch national Statistics Netherlands (1997-2007, 2001, 2006, 2010),* <br>*** **Unit of Analysis***: Wijk (neighborhood*	**N/A**	**N/A**

**Barcelona**(Spain)	2016	**PC_NVULN16T**<br>*"Percentage of non-vulnerable residents per nationality (**GNIMM16T/VULTOT11T**)"*<br><br>**GNIMM16T:**<br>*NON-vulnerable countries per nationality, EU27, USA, Canada, Norway, Luxembourg, Andorra, island, Litchestein*<br><br>**VULTOT16T:**<br>*total population (GSIMM01T+GNIMM01T)*<br><br>*****<br>**Data source:** *For age, profession, education, vulnerability: Instituto Nacional de Estadística (1991, 2001, 2011) and register of inhabitants (1996 or 1997, 2006, 2016)*<br><br>*****<br>**Unit of Analysis***: Census tract, Small Research Area or Neighborhood depending on year and variable*	**PC_UNI16T**<br>*"Percentage of people with university degree or higher (**UNI16T/EDUTOT16T**)"*<br><br>**UNI11T**:<br>*University studies OR CFGS higher degree*<br><br>**EDUTOT16T:**<br>*total population for education variable*<br><br>*****<br>**Data source:** *For age, profession, education, vulnerability: Instituto Nacional de Estadística (1991, 2001, 2011) and register of inhabitants (1996 or 1997, 2006, 2016)*<br><br>*****<br>**Unit of Analysis***: Census tract, Small Research Area or Neighborhood depending on year and variable*	**INCOME16T**<br><br>**RFDVALUE16R**: *Level of people considered high income*<br><br>*****<br>**Data source:** *For age, profession, education, vulnerability: Instituto Nacional de Estadística (1991, 2001, 2011) and register of inhabitants (1996 or 1997, 2006, 2016)*<br><br>*****<br>**Unit of Analysis***: Census tract, Small Research Area or Neighborhood depending on year and variable*	**RENT16T**<br><br>**RENTSM16ZEG:**<br>*Rent price per month in Euro/m2 for second hand renters.*<br><br>*****<br>**Data source:** *For age, profession, education, vulnerability: Instituto Nacional de Estadística (1991, 2001, 2011) and register of inhabitants (1996 or 1997, 2006, 2016)*<br><br>*****<br>**Unit of Analysis***: Census tract, Small Research Area or Neighborhood depending on year and variable*	**N/A**	**N/A**
**Copenhagen**(Denmark)	2015	**PC_NVUL15R**<br>*"Percentage of non-vulnerable community (**NVUL15R/VULNTOT15R**)"*<br><br>**NVUL15R:**<br>*Population with origins in Denmark or other Western countries*<br><br>**VULTOT15R:**<br>*Total of people answering vulnerability question*	**PC_UNI15R**<br>*"Percentage of residents with a university degree (**UNI15R/EDUTOT15R**)"*<br><br>**UNI15R:**<br>*Population with a university degree or higher*<br><br>**EDUTOT15R:**<br>*Total education*	**PC_HISES15R**<br>**"***Percentage of residents with high-income* **(HISES15R/SESTOT15R)***"*<br><br>**HISES15R:**<br>*High income*<br><br>**SESTOT15R:**<br>*Aggregate of all high income*<br><br>*****	**N/A**	**PC_PUBHO15R**<br>*"Percentage of residents not below poverty level (**PUB09R/TOTHH09R**)*<br><br>**PUB15R:**<br>*Those living in public housing*<br><br>**TOTHH15R:**<br>*Total household (denominator for housing)*	**N/A**

		<br>**Data source:** *Danish Statistical Office (yearly from 1990 to 2016)*<br><br><br>**Unit of Analysis***: Roder*	***<br>**Data source:** *Danish Statistical Office (yearly from 1990 to 2016)*<br><br>***<br>**Unit of Analysis***: Roder*	**Data source:** *Danish Statistical Office (yearly from 1990 to 2016)*<br><br>***<br>**Unit of Analysis***: Roder*		***<br>**Data source:** *Danish Statistical Office (yearly from 1990 to 2016)*<br><br>***<br>**Unit of Analysis***: Roder*	
**Dublin** (Ireland)	2016	**PC_NVULN16E**<br>*"Percentage of non-vulnerable community* ***(NVULN16E/VULTOT16E)****"*<br><br>**NVULN16E:**<br>*Non-vulnerable community; White Irish*<br><br>**VULTOT16E:**<br>*Total number of people answering the vulnerability question; Total Persons surveyed 'usually resident population by ethnic or cultural background'*<br><br>***<br>**Data source:** *Ireland Census (1996, 2002, 2006, 2011, 2016)*<br><br>***<br>**Unit of Analysis***: Electoral Divisions (called SAPS in 1996)*	**PC_UNI16E**<br>*"Percentage of residents with a university degree or higher (****UNI16E/EDUTOT16E****)"*<br><br>**UNI16E:**<br>*People with a university degree or higher; aggregate of various degrees and qualifications*<br><br>**EDUTOT16E:**<br>*Population over 15yo by highest level of education completed*<br><br>***<br>**Data source:** *Ireland Census (1996, 2002, 2006, 2011, 2016)*<br><br>***<br>**Unit of Analysis***: Electoral Divisions (called SAPS in 1996)*	**N/A**	**N/A**	**PC_NPOV16E**<br>*"Percentage of households that live in social housing* ***(SHOU11E/HHTOT11E)"***<br><br>**SHOU16E:**<br>*Number of households that live in social housing*<br><br>**HHTOT16E:**<br>*Total number of households; Total households surveyed on 'Permanent private households by type of occupancy'. All owners, all renters, and all 'living rent-free'/'occupied free of rent'/'not stated'*<br><br>***<br>**Data source:** *Ireland Census (1996, 2002, 2006, 2011, 2016)*<br><br>***<br>**Unit of Analysis***: Electoral Divisions (called SAPS in 1996)*	**PC_PROF16E**<br>***"****Percentage of people working in a professional occupation (****PROF16E/PROFTOT16E****)"*<br><br>**PROF16E:**<br>*People working in a professional occupation; Aggregate of (1) Employers & Managers and (2) Higher professional*<br><br>**PROFTOT16E:**<br>*Total occupation; Total persons; Occupation total denominator*<br><br>***<br>**Data source:** *Ireland Census (1996, 2002, 2006, 2011, 2016)*<br><br>***<br>**Unit of Analysis***: Electoral Divisions (called SAPS in 1996)*
**Valencia**(Spain)	2016	**PC_NVULN16T**<br>*"Percentage of non-vulnerable community* ***(NVULN16T/VULTOT01T)***<br><br>**NVULN16T**:	**PC_UNI16T**<br>*"Percentage of people with a university degree or higher* ***(UNI16T/EDUTOT16T)***	**N/A**	**N/A**	**N/A**	**N/A**

		Countries of birth: Countries in Africa, Philippines, Peru, Pakistan, Bolivia, Ecuador, Colombia, or the Dominican Republic<br><br>**VULTOT16T**:<br>*Total population*<br><br>***<br>**Data source:** *For age, profession, education, vulnerability: Instituto Nacional de Estadística censuses (1991, 2001, 2011) and register of inhabitants (1996, 2006, 2016).*<br><br>***<br>**Unit of Analysis***: Census tract or neighborhood depending on year and variable*	**UNI16T:**<br>*People with a university degree or higher in smallest geography*<br><br>**EDUTOT16T:**<br>*Total population*<br><br>**<br>**Data source:** *For age, profession, education, vulnerability: Instituto Nacional de Estadística censuses (1991, 2001, 2011) and register of inhabitants (1996, 2006, 2016).*<br><br>***<br>**Unit of Analysis***: Census tract or neighborhood depending on year and variable*				
**Vienna** (Austria)	2014/2015	**N/A**	**PC_UNI14T**<br>*"Percentage of residents with university degree (**UNI14T/EDUTOT14T**)"*<br><br>**UNI14T:**<br>*All people with university degree*<br><br>**EDUTOT14T:**<br>*Total number of all categories (total population)*<br><br>***<br>**Data source:** *For age, education, profession, socioeconomic status, and vulnerability: City of Vienna Austrian Public Data Portal (1991, 2001, 2014 or 2015 or 2016)*<br><br>***<br>**Unit of Analysis***: Zählgebiet (Sub-Districts)*	**PC_HISES15T**<br>*"Percentage of residents with high income (**HISES15T/SESTOT15T**)"*<br><br>**HISES15T:**<br>*residents in Sector groups: D,E,J,K,L,S,M,N,O,P,Q,R,U (high socioeconomic status)*<br><br>**SESTOT15T:**<br>*Total number of call categories (total population)*<br><br>***<br>**Data source:** *For age, education, profession, socioeconomic status, and vulnerability: City of Vienna Austrian Public Data Portal (1991, 2001, 2014 or 2015 or 2016)*<br><br>***<br>**Unit of Analysis***: Zählgebiet (Sub-Districts)*	**N/A**	**N/A**	**PC_PROF14T**<br>*"Percentage of residents with professional occupation (**PROF14T/PROFTOT14T)***<br><br>**PROF14T:**<br>*People working in professional education*<br><br>**PROFTOT14T:**<br>*Total number of all categories (total population)*<br><br>***<br>**Data source:** *For age, education, profession, socioeconomic status, and vulnerability: City of Vienna Austrian Public Data Portal (1991, 2001, 2014 or 2015 or 2016)*<br><br>***<br>**Unit of Analysis***: Zählgebiet (Sub-Districts)*

Timeframes

As shown above, data was collected at the smallest interval available for each country/city between 1990 and 2016, our final year of available data collection. Thus, time intervals varied from annual (Copenhagen) to every 10 years (the US, UK, Canada), and in some cases, less regular intervals (i.e.for Lyon and Nantes, 1999 followed by 2006). In some cases, the years for which data was available from different sources for cities varied between sources. In these cases, in the final dataset, we used the year for which the majority of data was collected and merged data from other sources for the closest possible year to form datasets for Time 1, Time 2, and Time 3 that correspond as close as possible with the 1990s, 2000s, and 2010s.

Data Processing

Calculating Relative Values

Once data was collected for each city, we standardized the measurement of each variable for each time and geographic unit by calculating the percentage of the population in each geographic area represented by the variable. For example, we calculated the percent of each geographic area that had a low socioeconomic status, were a member of a racial or ethnic minority, etc.

Standardizing geographic scale: the CUDA Approach

In most cases, the small-area geographies at which data was colected changed across years. It is important to note, though, that we were only analyzing data within the formal city boundaries of our 28 cities. Thus, there was usually not a substantial degree of change. In order to standardize the boundaries for analysis across time, we used a hierarchically ordered set of techniques – an approach we call CUDA. CUDA is an acronym for the hierarchy, which stands for Crosswalk, Unsplit, Dasymetric reapportionment, and aereal reapportionment. In essence, we used this hierarchy to define our order of preference for how to deal with changes in boundaries across time. We took this approach because the overall goal was to develop the most accurate reflection of the data rather tan one standard approach. Where available, we first sought to employ “crosswalks” provided by the public agencies that managed the data to explain how reallocation across years was done and allow for a standardized geography to be reconstructed. When no crosswalk was available, we next sought to “unsplit” the geographies. In many cases, the inconsistencies across time were simply a matter of larger areas being subdivided. In order to create standard boundaries, we unsplit these areas and used the larger geography for analysis. For some cities, we had to turn to reapportioning techniques in order to complete the standardization. When reapportioning, we first used a dasymetric approach wherein aerial imagery or land use files formed the basis for identifying populated areas before data was reapportioned to new areas across years. While this method does not eliminate error, it does reduce it. Finally, when necessary, we employed an areal reapportionment with manual adjustments for outliers. These manual adjustnments were carefully considered, often through a tract-by-tract check in the case of all U.S. cities.

Real estate development dataset

This dataset was built as part of the EU-funded Greenlulus project (2016-2021, GA670834) in 2016-2018, collected to describe the number of new residential properties built per time period. Depending on the city, two types of datasets were available: 1) the year specific residential properties were built (22 cities) and 2) new residential developments per spatial unit per time period collected (six cities). The datasets were available from cadastral, tax or building permit data, provided either through free access online, upon emailing the city in question or in a handful of cases provided by a fee from the city in question.

Most of the data required processing before use, for example, removing non-residential properties. All data was spatialized by either by mapping X,Y coordinates or joining appropriate data columns to shapefiles provided by the city. Ultimately the aim was for each tract to have data for the total number of new residential developments in every year.

Supplementary Table 3 provides details on the type of dataset used per city.

Supplementary Table 3. Development data collected per city

City	Type of data	Data details
Amsterdam	Year built	The Cadaster of Amsterdam holds the BAG (Basisregistraties adressen en gebouwen, Basic Registration of Addresses and Buildings in English). A fee was paid to access the Cadaster and the BAG extract to obtain the year built information for all buildings in Amsterdam.
Atlanta	Year built	Annual city tax roll contains year built data for all building types, collected by the Fulton County Board of Assessors. Available free of charge online.
Austin	Year built	The land database digital map, created by the City of Austin Development Services Department, provides year built information. Available free of charge online.
Baltimore	Year built	Real property shapefile available for free of charge download online.
Barcelona	Year built	Cadastral parcels and year built was downloaded from the INSPIRE project at the Dirección General del Catastro, government of Spain. Available free of charge online.
Boston	Year built	Tax parcel information contains building ages, derived from the City of Boston's Building Permits data base thanks to collaboration between the Boston Area Research Initiative and the City of Boston. Available free of charge online.
Bristol	New residential developments per spatial unit	The Consumer Data Research Centre provided residential dwelling ages, grouped into approximately 10-year age bands and provided per spatial unit (263 in Bristol). We used four age bands to assess new building construction: 1) 1983-1992; 2) 1993-1999; 3) 2000-2009; 4) 2010-2015. Data provides the numbers of properties in each Lower Super Output Area (LSOA, around 1000 properties) for each age band, with data rounded at source near 10. Available free of charge online.

Calgary	Year built	The City of Calgary provided building ages in tabular data form from 2014-2018. From 1999 to 2013, permit history was used, with the Geodatabase files allowing for every building permit to be spatially correlated to actual parcels. Data was provided via email by the City of Calgary.
Cleveland	Year built	Parcel data files from Cuyahoga county provides the year all properties were built in Cleveland. Data was provided via email by the City of Cleveland.
Copenhagen	Year built	Cadastral data contains year built information, gathered by city of Copenhagen. Data was provided via email by Lifa, a Danish property inspection company.
Denver		Parcel data files from Denver Open Data Catalogue provide year built information. Available free of charge online.
Detroit	Year built	Parcel data files from City of Denver Open Data Portal provides year built information. Available free of charge online.
Dublin	Data not available	Data not available
Edinburgh	New residential developments per spatial unit	New buildings built per year from 2004 to 2016 per 2001 Data Zone (549 Data Zones in Edinburgh), provided by Scottish Neighbourhood Statistics. Calculated increase in total dwellings from year to year in overall dwellings per Data Zone and in number of dwellings per tax band.
Louisville	Year built	Building permits issued or in progress provides data on new buildings under construction, collected by the Louisville Office of Construction Review and posted on Louisville Open Data. Available free of charge online.
Lyon	New residential developments per spatial unit	Data provided by the National Institute of Statistics and Economic Studies (INSEE) lists new buildings built between 1991-2005 and per year from 2006 to 2010. Partial data per spatial unit (IRIS, 174 in Lyon) only available between 2011 to 2015. Available free of charge online, registration required on the Reseau Quetelet data platform for social scientists.
Montreal	Year built	Two data sets were combined to provide a comprehensive view of the city. 1) Building age data provided by the Canada Broadcast Corporation (CBC) for downtown area and 2) 2016 census data from Statistics Canada provided data on occupied private dwellings by period of construction (1960 or before; 1961-1980; 1981-1990; 1991-2000; 2001-2005; 2006-2010; 2011-2016). Available free of charge online.
Milwaukee	Year built	Master property file from Milwaukee Open Data provided year built information. Available free of charge online.
Nantes	New developments per spatial unit	Data provided by the National Institute of Statistics and Economic Studies (INSEE) lists new buildings built between 1991-2005 and per year from 2006 to 2010. Partial data per spatial unit (IRIS, 91 in Nantes) only available between 2011 to 2015. Available free of charge online, registration required on the Reseau Quetelet data platform for social scientists.
Portland	Year built	Building permits issued or in progress provides data on new buildings under construction, collected by the Portland Development Services Center since 1995. Available free of charge online. https://www.portlandmaps.com/arcgis/rest/services/Public/COP_OpenData_PlanningDevelopment/MapServer/89
Philadelphia	New developments per spatial unit	Building permits issued for new residential construction available free of charge online.

San Francisco	Year built	DataSF provides year built information, collected by the San Francisco property tax assessor. Available free of charge online.
Seattle	Year built	Building permits issued or in progress provides data on new buildings under construction, from data collected by the Seattle Department of Constructions and Inspections. Available free of charge online.
Sheffield	New residential developments per spatial unit	The Consumer Data Research Centre provided residential dwelling ages, grouped into approximately 10-year age bands and provided per spatial unit (345 in Sheffield). We used four age bands to assess new building construction: 1) 1983-1992; 2) 1993-1999; 3) 2000-2009; 4) 2010-2015. Data provides the numbers of properties in each Lower Super Output Area (LSOA, around 1000 properties) for each age band, with data rounded at source near 10. Available free of charge online.
Valencia	Year built	Cadastral parcels and year built was downloaded from the INSPIRE project at the Dirección General del Catastro, government of Spain. Available free of charge online.
Vancouver	Year built	Property tax report table from city of Vancouver provides data on building age. Available free of charge online.
Vienna	New residential developments per spatial unit	City of Vienna, Department for Economy, Labour and Statistics, provided data on the number of constructed residential buildings per year at the Zählbezirk (smallest geography) spatial unit level. Data is collected by the Building-Housing-Address-Register of the Austrian Statistic Institute. Data was provided via email from the City of Vienna.
Washington DC	Year built	Historical data on the buildings of Washington DC, collected over 15 years by Brian Kraft, with support from JMT Inc., for the DC Historic Preservation Office. Available free of charge online.

Supplementary References

1. Atkinson, R. Measuring gentrification and displacement in Greater London. *Urban Studies* ***37*****(1)**, 149-165 (2000).
2. Brueckner, Jan K, and Stuart S Rosenthal. Gentrification and neighborhood housing cycles: will America's future downtowns be rich? *The Review of Economics and Statistics* **91(4)**, 725-743 (2009).
3. Clark, E. The order and simplicity of gentrification: a political challenge. In Atkinson, R. and Bridge, G. (eds.) *Gentrification in a global context: the new urban colonialism* 261-269 (Rouledge Press, London, 2005).
4. Easton, S., Lees, L., Hubbard, P., & Tate, N. Measuring and mapping displacement: The problem of quantification in the battle against gentrification. *Urban studies,* ***57*****(2)**, 286-306 (2020).
5. Elliott-Cooper, A., Hubbard, P., & Lees, L. Moving beyond Marcuse: Gentrification, displacement and the violence of un-homing. *Progress in Human Geography,* ***44*****(3)**, 492-509 (2020).
6. Freeman, L., & Braconi, F. Gentrification and displacement New York City in the 1990s. *Journal of the American Planning Association*, ***70*****(1)**, 39-52 (2004).
7. Glass, R. *London: aspects of change, No. 3* (MacGibbon & Kee, London,1964).
8. Glick, J. Gentrification and the racialized geography of home equity. *Urban Affairs Review*, ***44*****(2)**, 280-295 (2008).
9. Hamnett, C. The blind men and the elephant: the explanation of gentrification. *Transactions of the institute of British Geographers*, **16(2)**, 173-189 (1991).
10. Hammel, D. J., & Wyly, E. K. A model for identifying gentrified areas with census data. *Urban Geography*, ***17*****(3)**, 248-268 (1996).
11. Hwang, J., & Sampson, R. J. Divergent pathways of gentrification: Racial inequality and the social order of renewal in Chicago neighborhoods. *American Sociological Review*, ***79*****(4)**, 726-751 (2014).
12. Kahn, Matthew E, and Randall Walsh. Cities and the Environment. *Handbook of regional and urban economics 5*, 405-465 (Elsevier, North Holland, 2015).
13. Janoschka, M., Sequera, J., & Salinas, L. Gentrification in Spain and Latin America—a Critical Dialogue. *International journal of urban and regional research*, ***38*****(4)**, 1234-1265 (2014).
14. Lees, L., Slater, T., & Wyly, E. *Gentrification* (Routledge, London and New York, 2008).
15. Ley, D. Inner-city revitalization in Canada: A Vancouver case study. *The Canadian Geographer/Le Géographe Canadien*, ***25*****(2)**, 124-148 (1981).
16. Ley, D. Alternative explanations for inner-city gentrification: a Canadian assessment. *Annals of the association of american geographers*, ***76*****(4)**, 521-535 (1986).
17. Marcuse, P. Gentrification, abandonment, and displacement: Connections, causes, and policy responses in New York City. *Wash. UJ Urb. & Contemp. L.*, ***28***, 195-240 (1985).
18. Newman, K., & Wyly, E. K. The right to stay put, revisited: Gentrification and resistance to displacement in New York City. *Urban studies*, ***43*****(1)**, 23-57 (2006).
19. Slater, T. Gentrification of the city. *The New Blackwell companion to the city*, *1* (John Wiley & Sons Press, New York, 2011).
20. Smith, N. Toward a theory of gentrification as back to the city movement by capital, not people. *Journal of the American planning association*, ***45*****(4)**, 538-548 (1979).

21. Zook, M., Shelton, T., & Poorthuis, A. Big data and the city. In *Handbook of Urban Geography*. (Edward Elgar Publishing, Chelthanum, UK, Northampton, MA, 2019).
