# Supplementary material for: Green gentrification in European and North American cities
Source: Nat Commun. 2022 Jul 2;13:3816. doi: 10.1038/s41467-022-31572-1 (PMC9250502; doi:10.1038/s41467-022-31572-1)
Supplement: Supplementary file 3 — Description of Additional Supplementary Information [file 41467_2022_31572_MOESM3_ESM.pdf]

Description of Additional Supplementary Information

Title: Supplementary Data

Description: In the results reported, the value of p corresponds to the probability that the parameter is greater than 0 and so, large values imply relevant positive effects while small values imply relevant negative effects. The variable codes used in the supplementary data file are: CH_GSAB_PRE2: Changes in greenspace prior to Period 2 CH_GSAB_PRE3: Changes in greenspace prior to Period 3 CENTER: Distance from the centroid of the tract to the approximate centerpoint of the historic city center TRACTDENSITY_2010:

Residential density of the tract in 2010 PRE_1990_GREEN: Percent of area of the tract covered in greenspace prior to 1990 NEWTRANSITB_PRE2: Number of new transit stops added to the tract prior to Period 2 NEWTRANSITB_PRE3: Number of new transit stops added to the tract prior to Period 3 DEV_TIME2: Number of new residential buildings constructed in the tract during Period 2 DEV_TIME3: Number of new residential buildings constructed in the tract during Period 3 DEV_TIME23: Number of new residential buildings constructed in the tract during Periods 2 and 3
