# Supplementary material for: Green gentrification in European and North American cities
Source: Nat Commun. 2022 Jul 2;13:3816. doi: 10.1038/s41467-022-31572-1 (PMC9250502; doi:10.1038/s41467-022-31572-1)
Supplement: Supplementary file 5 — Reporting Summary [file 41467_2022_31572_MOESM5_ESM.pdf]

nature portfolio

Corresponding author(s): Isabelle Anguelovski

Last updated by author(s): 2022-30-05

Reporting Summary

Nature Portfolio wishes to improve the reproducibility of the work that we publish. This form provides structure for consistency and transparency in reporting. For further information on Nature Portfolio policies, see our Editorial Policies and the Editorial Policy Checklist.

Statistics

For all statistical analyses, confirm that the following items are present in the figure legend, table legend, main text, or Methods section.

n/a	Confirmed	
[x]	[ ]	The exact sample size (n) for each experimental group/condition, given as a discrete number and unit of measurement
[x]	[ ]	A statement on whether measurements were taken from distinct samples or whether the same sample was measured repeatedly
[x]	[ ]	The statistical test(s) used AND whether they are one- or two-sided<br>*Only common tests should be described solely by name; describe more complex techniques in the Methods section.*
[x]	[ ]	A description of all covariates tested
[x]	[ ]	A description of any assumptions or corrections, such as tests of normality and adjustment for multiple comparisons
[x]	[ ]	A full description of the statistical parameters including central tendency (e.g. means) or other basic estimates (e.g. regression coefficient) AND variation (e.g. standard deviation) or associated estimates of uncertainty (e.g. confidence intervals)
[x]	[ ]	For null hypothesis testing, the test statistic (e.g. F, t, r) with confidence intervals, effect sizes, degrees of freedom and P value noted<br>*Give P values as exact values whenever suitable.*
[ ]	[x]	For Bayesian analysis, information on the choice of priors and Markov chain Monte Carlo settings
[x]	[ ]	For hierarchical and complex designs, identification of the appropriate level for tests and full reporting of outcomes
[x]	[ ]	Estimates of effect sizes (e.g. Cohen's d, Pearson's r), indicating how they were calculated

Our web collection on statistics for biologists contains articles on many of the points above.

Software and code

Policy information about availability of computer code

Data collection	https://overpass-turbo.eu was used to download openstreet map geospatial data
Data analysis	R 3.6.1 and the R-INLA package was used for statistical analysis, ArcGIS 10.1 was used for geospatial processing

For manuscripts utilizing custom algorithms or software that are central to the research but not yet described in published literature, software must be made available to editors and reviewers. We strongly encourage code deposition in a community repository (e.g. GitHub). See the Nature Portfolio guidelines for submitting code & software for further information.

Data

Policy information about availability of data

All manuscripts must include a data availability statement. This statement should provide the following information, where applicable:

- Accession codes, unique identifiers, or web links for publicly available datasets
- A description of any restrictions on data availability
- For clinical datasets or third party data, please ensure that the statement adheres to our policy

All non-proprietary data generated during the study and analyzed within the study will be available in public repository being developed by the Barcelona Lab for Urban Environmental Justice and Sustainability. This repository will be online. Until the online repository is fully developed, data is available upon reasonable request to the corresponding author, Isabelle Anguelovski.

Field-specific reporting

Please select the one below that is the best fit for your research. If you are not sure, read the appropriate sections before making your selection.

☐ Life sciences ☒ Behavioural & social sciences ☐ Ecological, evolutionary & environmental sciences

For a reference copy of the document with all sections, see nature.com/documents/nr-reporting-summary-flat.pdf

Behavioural & social sciences study design

All studies must disclose on these points even when the disclosure is negative.

Study description	Data is quantitative and spatial. See full supplementary materials. It includes green spatial data, demographic data, and real estate data. The study uses a spatially weighted Bayesian model to test the green gentrification hypothesis across 28 cities in 9 countries in North America and Europe. The Bayesian model is designed to measure the relationship between gentrification (dependent variable) and greening (independent variable) in the presence of covariates (new transit, new development, prior greening, distance to city center, city GDP, Country, region).
Research sample	The study analyzes a sample of 28 mid-sized (population between 500,000 and 1,500,000) cities in Europe and North America. The sample was selected based on an exhaustive review of all mid-sized cities in Western Europe, the United States and Canada. The review included a review of growth trajectories, greening trajectories and basic demographics. The sample was selected to maximize diversity of cities when considering data availability.
Sampling strategy	The study uses a purposive, non-probability sample in order to leverage available data and test the main hypothesis across as many contexts as possible.
Data collection	All demographic data was obtained from public data sources including governmental statistics offices or their representatives. All greenspace data was developed by the researchers through a combination of public geospatial files provided by cities and outreach to city staff members, archival document searches, aerial imagery analysis performed manually by the research team, media document analysis, and site visits. All other data was obtained from public online sources. Researchers were not blinded to the study hypotheses in the data search.
Timing	Data collection occurred between August 2016 and August 2019.
Data exclusions	No data was excluded from the analysis.
Non-participation	Original data collected did not involve participants. Participation rates are applicable for the governmental statistics used in the study can be found in the documentation for each government.
Randomization	Original data collected for this study did not involve participants.

Reporting for specific materials, systems and methods

We require information from authors about some types of materials, experimental systems and methods used in many studies. Here, indicate whether each material, system or method listed is relevant to your study. If you are not sure if a list item applies to your research, read the appropriate section before selecting a response.

Materials & experimental systems

n/a	Involved in the study	
☒	☐	Antibodies
☒	☐	Eukaryotic cell lines
☒	☐	Palaeontology and archaeology
☒	☐	Animals and other organisms
☒	☐	Human research participants
☒	☐	Clinical data
☒	☐	Dual use research of concern

Methods

n/a	Involved in the study	
☒	☐	ChIP-seq
☒	☐	Flow cytometry
☒	☐	MRI-based neuroimaging
